# Long-term prognosis after intracerebral haemorrhage

**DOI:** 10.1177/2396987320953394

**Published:** 2020-09-02

**Authors:** Koen M van Nieuwenhuizen, Ilonca Vaartjes, Jamie I Verhoeven, Gabriel JE Rinkel, L Jaap Kappelle, Floris HBM Schreuder, Catharina JM Klijn

**Affiliations:** 1 Department of Neurology and Neurosurgery, Brain Center Rudolf Magnus, University Medical Center Utrecht, Utrecht, the Netherlands; 2 Julius Center for Health Sciences and Primary Care, University Medical Center Utrecht, Utrecht, the Netherlands; 3 Department of Neurology, Donders Institute of Brain, Cognition and Behaviour, Center for Neuroscience, Radboud University Medical Center, Nijmegen, the Netherlands

**Keywords:** Intracerebral haemorrhage, long-term mortality, recurrent events, cardiovascular prognosis

## Abstract

**Introduction:**

The aim of this study was to determine the risk of recurrent intracerebral haemorrhage (ICH), ischaemic stroke, all stroke, any vascular event and all-cause mortality in 30-day survivors of ICH, according to age and sex.

**Patients and methods:**

We linked national hospital discharge, population and cause of death registers to obtain a cohort of Dutch 30-day survivors of ICH from 1998 to 2010. We calculated cumulative incidences of recurrent ICH, ischaemic stroke, all stroke and composite vascular outcome, adjusted for competing risk of death and all-cause mortality. Additionally, we compared survival with the general population.

**Results:**

We included 19,444 ICH-survivors (52% male; median age 72 years, interquartile range 61–79; 78,654 patient-years of follow-up). First-year cumulative incidence of recurrent ICH ranged from 1.5% (95% confidence interval 0.9–2.3; men 35–54 years) to 2.4% (2.0–2.9; women 75–94 years). Depending on age and sex, 10-year risk of recurrent ICH ranged from 3.7% (2.6–5.1; men 35–54 years) to 8.1% (6.9–9.4; women 55–74 years); ischaemic stroke 2.6% to 7.0%, of all stroke 9.9% to 26.2% and of any vascular event 15.0% to 40.4%. Ten-year mortality ranged from 16.7% (35–54 years) to 90.0% (75–94 years). Relative survival was lower in all age-groups of both sexes, ranging from 0.83 (0.80–0.87) in 35- to 54-year-old men to 0.28 (0.24–0.32) in 75- to 94-year-old women.

**Discussion:**

ICH-survivors are at high risk of recurrent ICH, of ischaemic stroke and other vascular events, and have a sustained reduced survival rate compared to the general population.

**Conclusion:**

The high risk of recurrent ICH, other vascular events and prolonged reduced survival-rates warrant clinical trials to determine optimal secondary prevention treatment after ICH.

## Introduction

Spontaneous intracerebral haemorrhage (ICH) is an important cause of global disease burden, accounting for 10% to 15% of strokes in high-income countries, and for 20% to 50% in developing countries.^1^ ICH has a 30-day case fatality of around 40%.^2^ Those who survive have an increased risk of recurrent ICH, which is higher after lobar than after non-lobar ICH.^3^ Two systematic reviews of the literature suggested that ICH survivors are also at high risk of ischaemic stroke and other thrombotic vascular events,^3,4^ but previous studies were limited by relatively small patient numbers, being hospital based, a short duration of follow-up, or a combination of these. Information on the proportional risks of haemorrhagic and ischaemic events has important implications for optimal secondary prevention, including decisions on whether or not to start or resume antithrombotic medication after ICH.

In a large, population-based record-linkage study, we assessed in patients who survived 30 days after ICH, the long-term risk of ICH recurrence, of ischaemic stroke and all stroke, of a composite vascular outcome, and of death, across difference age and sex strata.

## Patients and methods

### Data collection

We constructed a cohort of patients surviving 30 days after a first ICH through data linkage of the hospital discharge register (HDR), the population register (PR) and cause of death register (CDR) in the Netherlands. Registers and linkage procedures have been previously described in detail.^5,6^ In the period from 1998 to 2004, from all hospital admissions, 1.1% to 1.6% of the records were missing. Between 2005 and 2010, the percentage of missing records varied between 3.8% and 14% (Supplementary Table e-1). The data sources and method of data collection of the HDR, PR and CDR have remained unchanged during the study period.

The PR contains information of all Dutch citizens, including date of birth, sex, current address, ethnicity and marital status. The CDR contains information on the date of death and causes of death. The overall validity of these registers has been proven to be high.^7^ We linked the registries by a personal identifier using a combination of postal code, sex and date of birth to ensure all cases were unique. Approximately 85% of the Dutch population has a unique combination of these variables.^8^ The PR is available electronically since 1995; therefore linkage of the registries can be done from 1995 onwards.

### Cohort selection

We selected all patients aged between 35 and 94 years with a first admission for a principal diagnosis of ICH (International Classification of Diseases, ninth revision (ICD-9) code 431) from the HDR between 1 January 1998 and 31 December 2010. We excluded patients below 35 years of age because in those patients determinants of vascular outcomes are likely to be different than in older patients. The number of patients of 95 years and older was too small to allow meaningful analyses. We excluded patients who were admitted for ICH in the three years before the study period and those who died during admission or within 30 days after discharge. In a previous study, we showed high validity of the use of ICD-9 codes to identify patients with ICH.^6^ The presence and extent of co-morbidity were determined in the HDR using a modified Charlson Comorbidity Index (CCI), which is a valid and reliable method to measure co-morbidity in clinical research.^9^ The CCI ranges from 0 to 24 points, zero points representing no co-morbidity.

### Outcomes

Information about new events during follow-up for the outcomes ICH, ischaemic stroke, unspecified stroke, all stroke, the composite vascular outcome (including stroke, cardiac and peripheral vascular disease) and death was obtained from the HDR and CDR. ICD-9 and ICD-10 codes that represent the outcome events are listed in Supplementary Table e-2; all outcomes were based on principal diagnosis. Admissions within 30 days after discharge from the admission for the index ICH were discarded as these include transfers from one hospital to another without occurrence of a new event as well as early re-admissions without a new event as a result of morbidity due to the index ICH.^10^ Second and beyond events (e.g. a second ischaemic stroke) were disregarded.

### Data analysis

We divided patients into three age groups, each spanning 20 years (35–54, 55–74 and 75–94 years) and by sex.

We performed competing risk analyses for death to calculate the cumulative incidence of ICH, ischaemic stroke, unspecified stroke, all stroke and the composite vascular outcome at 1, 2, 5 and 10 years, with 95% confidence intervals (CIs),^11^ and calculated the cumulative incidence of death with 95% CIs. In addition, we performed multivariable Cox proportional hazard regression for all outcomes to obtain hazard ratios with 95% CIs for the co-variables age and sex.

To determine the effect of the index ICH on the mortality of ICH survivors, we compared mortality between ICH survivors and the general Dutch population, matched for age, sex and calendar year, obtained from the Human Mortality Database (www.mortality.org) and divided the observed mortality by the expected mortality.

All statistical analyses were performed in SAS/STAT 12.1 (SAS Institute, Cary, NC, USA) and R 3.0.2 (R Foundation for Statistical Computing, Vienna, Austria) using the packages cmprsk, survival and relsurv.

### Ethical approval

The University Medical Center Utrecht Medical Ethics Review Committee assessed the protocol and waived the requirement for ethical review. We performed all analyses in a secured environment of Statistics Netherlands according to the Dutch privacy legislation.

## Results

We identified 41,950 patients who had experienced a first ICH. After exclusion of 838 patients who were younger than 35 years, 299 patients who were 95 or older, 3116 patients who died before reaching the hospital and 18,253 patients who did not survive beyond 30 days, a total of 19,444 patients fulfilled our inclusion criteria. Median age was 72 years (interquartile range (IQR) 61–79) and 52% were men. Median duration of follow-up was 3.2 years (IQR 1.2–6.2). The total number of patient-years of follow-up was 78,654. Characteristics of the cohort are summarized in Table 1.

**Table 1.** Characteristics, admission setting and -duration, and duration of follow-up of 30-day ICH survivors overall, and according to age and sex.

	All	Men 35–54 years	Men 55–74 years	Men 75–94 years	Women 35–54 years	Women 55–74 years	Women 75–94 years
Total, n	19,444	1478	5520	3450	1196	3508	4592
Median age, years (IQR)	72 (61–79)	48 (44–52)	66 (61–71)	80 (77–83)	48 (44–52)	67 (61–71)	81 (78–85)
Men, n (%)	10,148 (52.2%)						
Academic hospital, n (%)	2798 (14.4%)	405 (27.4%)	761 (14.6%)	330 (9.6%)	307 (25.7%)	523 (14.9%)	472 (10.3%)
Median duration admission, days (IQR)	15 (8–28)	12 (6–24)	14 (7–25)	16 (9–29)	13 (7–24)	15 (8–27)	18 (10–32)
CCI > 0, n (%)	5896 (30.3%)	300 (20.3%)	1706 (32.7)	1332 (38.6%)	238 (19.9%)	941 (26.8%)	1379 (30.0%)
Married or living together, n (%)	10,047 (51.7%)	862 (58.3%)	3760 (72.0%)	2107 (61.1%)	754 (63.0%)	1761 (50.2%)	803 (17.5%)
Native Dutch, n (%)	16,553 (85.1%)	1140 (77.1%)	4319 (82.7%)	3140 (91.0%)	938 (78.4%)	2933 (83.6%)	4083 (88.9%)
Median follow-up, years (IQR)	3.2 (1.2–6.2)	4.9 (2.1–8.4)	4.0 (1.6–6.9)	2.2 (0.9–4.2)	5.3 (2.3–8.8)	4.0 (1.7–7.3)	2.3 (0.8–4.5)
Follow-up ≥2 years, n (%)	12,639 (65.0%)	1157 (78.3%)	3701 (67.1%)	1818 (52.7%)	962 (80.4%)	2520 (71.8%)	2481 (54.0%)
Follow-up ≥10 years, n(%)	1478 (7.6%)	245 (16.6%)	456 (8.3%)	63 (1.8%)	230 (19.2%)	369 (10.5%)	115 (2.5%)

n: number; IQR: interquartile range; CCI: Charlson Comorbidity Index.

### Outcomes

During follow-up, 859 patients (4.4%) were diagnosed with recurrent ICH (507 of these were fatal), 812 patients (4.2%) with ischaemic stroke (fatal in 162) and 1968 patients (10.1%) experienced a stroke that was not further specified as ischaemic or haemorrhagic (fatal in 437). The outcome all stroke occurred in 3377 patients (17.4%, fatal in 1385; Supplementary Table e-3). The composite vascular outcome occurred in 4965 patients (25.5%, fatal in 2433; Supplementary Table e-4). A total of 8239 patients (42.4%) died from any cause.

One-year, 2-year, 5-year and 10-year cumulative risks and their 95% CIs for the various outcomes in the three age-groups for men and women are summarized in Table 2.

**Table 2.** Cumulative risks with 95% confidence intervals of recurrent intracerebral haemorrhage, ischaemic stroke, unspecified stroke, all stroke, the composite vascular outcome and all-cause death according to age and sex.

	1 year	2 years	5 years	10 years
	Recurrent intracerebral haemorrhage			
Men				
35–54	1.5 (1.0–2.3)	1.8 (1.2–2.6)	2.7 (1.9–3.7)	3.7 (2.6–5.1)
55–74	1.7 (1.4–2.1)	2.5 (2.1–3.0)	4.2 (3.6–4.8)	6.6 (5.8–7.6)
75–94	2.2 (1.8–2.8)	2.8 (2.3–3.4)	4.4 (3.7–5.2)	5.2 (4.4–6.2)
Women				
35–54	1.5 (0.9–2.3)	1.7 (1.0–2.5)	3.0 (2.1–4.2)	3.9 (2.7–5.4)
55–74	2.0 (1.6–2.5)	2.8 (2.3–3.4)	4.8 (4.0–5.6)	8.1 (6.9–9.4)
75–94	2.4 (2.0–2.9)	3.1 (2.6–3.7)	4.6 (4.0–5.3)	5.5 (4.7–6.3)
	Ischaemic stroke			
Men				
35–54	0.5 (0.2–1.0)	0.9 (0.5–1.6)	1.5 (0.9–2.3)	3.1 (2.1–4.5)
55–74	1.6 (1.2–1.9)	2.5 (2.1–2.9)	4.6 (4.0–5.3)	7.0 (6.1–7.9)
75–94	1.7 (1.3–2.2)	2.7 (2.1–3.3)	4.4 (3.7–5.2)	5.9 (4.9–6.9)
Women				
35–54	0.7 (0.3–1.3)	0.9 (0.5–1.6)	1.8 (1.1–2.8)	2.6 (1.6–4.0)
55–74	1.1 (0.8–1.5)	2.3 (1.8–2.8)	4.7 (4.0–5.6)	6.6 (5.6–7.8)
75–94	1.5 (1.2–1.9)	2.6 (2.2–3.1)	4.6 (4.0–5.3)	5.6 (4.8–6.5)
	Unspecified stroke			
Men				
35–54	0.4 (0.2–0.9)	0.9 (0.5–1.5)	1.9 (1.2–2.8)	2.9 (1.9–4.2)
55–74	2.6 (2.1–3.0)	4.0 (3.4–4.5)	7.6 (6.8–8.4)	11.2 (10.1–12.3)
75–94	6.7 (5.9–7.6)	9.9 (8.9–11.0)	14.6 (13.3–15.9)	17.7 (16.2–19.3)
Women				
35–54	0.9 (0.5–1.6)	1.3 (0.8–2.2)	2.8 (1.9–4.1)	4.3 (2.9–6.1)
55–74	2.4 (1.9–3.0)	3.6 (3.0–4.2)	7.6 (6.6–8.6)	12.1 (10.6–13.6)
75–94	7.7 (6.9–8.5)	11.0 (10.1–12.0)	17.7 (16.5–19.0)	20.4 (19.0–21.8)
	All stroke			
Men				
35–54	2.8 (2.0–3.7)	4.2 (3.3–5.4)	6.3 (5.0–7.7)	9.9 (0.8–12.1)
55–74	5.7 (5.1–6.3)	8.5 (7.8–9.4)	15.2 (14.1–16.3)	22.2 (20.7–23.7)
75–94	10.2 (9.2–11.3)	14.5 (13.3–15.7)	21.4 (19.9–22.9)	26.2 (24.4–28.1)
Women				
35–54	3.3 (2.4–4.4)	4.3 (3.3–5.7)	7.9 (6.3–9.8)	10.4 (8.3–12.8)
55–74	5.7 (4.9–6.5)	8.6 (7.7–9.6)	15.7 (14.4–17.1)	23.9 (21.9–25.8)
75–94	10.2 (10.5–12.4)	14.5 (15.2–17.4)	15.7 (24.2–27.0)	23.9 (28.0–31.2)
	Composite vascular outcome			
Men				
35–54	4.6 (3.6–5.8)	7.2 (5.9–8.6)	12.0 (8.9–12.8)	20.0 (17.2–22.9)
55–74	9.2 (8.5–10.1)	14.0 (13.1–15.0)	25.2 (23.9–26.6)	37.7 (35.9–39.6)
75–94	14.6 (13.4–15.9)	21.9 (20.4–23.3)	33.4 (31.7–35.1)	40.4 (38.3–42.4)
Women				
35–54	4.3 (3.2–5.5)	6.0 (4.4–7.1)	10.8 (8.9–12.8)	15.0 (12.4–17.8)
55–74	7.4 (6.5–8.3)	12.0 (10.9–13.1)	21.8 (20.3–23.4)	33.7 (31.5–35.8)
75–94	14.1 (13.1–15.1)	20.5 (19.3–21.8)	32.2 (30.4–33.7)	39.0 (37.2–40.8)
	All-cause death			
Men				
35–54	4.7 (3.7–5.9)	7.0 (5.8–8.5)	12.2 (10.4–14.2)	20.2 (17.4–23.4)
55–74	10.5 (9.7–11.4)	16.0 (15.0–17.0)	31.6 (30.2–33.0)	56.8 (54.7–58.9)
75–94	25.0 (23.6–26.5)	38.9 (37.3–40.6)	67.1 (65.3–68.9)	90.0 (88.2–91.6)
Women				
35–54	4.1 (3.1–5.4)	6.1 (4.8–7.6)	10.6 (8.8–12.7)	16.7 (14.1–19.8)
55–74	9.0 (8.1–10.0)	14.1 (13.0–15.4)	29.0 (27.3–30.8)	48.8 (46.4–51.3)
75–94	24.5 (23.3–25.8)	36.1 (34.7–37.5)	63.8 (62.2–65.4)	87.4 (85.7–89.0)

### Risk of outcomes according to age and sex

In patients within the age groups 55 to 74 and 75 to 94 years, for those patients in whom the type of stroke was specified, the cumulative risk of ischaemic stroke was comparable to the risk of recurrent ICH for all time periods of follow-up, whereas in patients aged 34 to 54, the risk of recurrent ICH was higher than that of ischaemic stroke (Table 2). The rate of unspecified stroke was substantial in all age groups in men and in women, most prominently in the age groups 55 to 74 years and 75 to 94 years (Table 2).

Older age was associated with a higher risk of all outcomes (Table 3). Men had a higher risk of ischaemic stroke, of the composite vascular outcome and of death than women (Table 3). We found no statistically significant differences between men and women in the occurrence of recurrent ICH, ischaemic stroke, unspecified stroke or all stroke.

**Table 3.** Hazard ratios with 95% confidence intervals of the various outcomes according to age and sex.

	Recurrent ICH	Ischaemic stroke	Unspecified stroke	All stroke	Composite vascular outcome	Death
Age^a^	1.02 (1.02–1.03)	1.03 (1.03–1.04)	1.07 (1.07–1.08)	1.04 (1.04–1.05)	1.04 (1.04–1.05)	1.08 (1.07–1.08)
Male sex	0.96 (0.84–1.10)	1.16 (1.01–1.33)	1.00 (0.91–1.10)	0.99 (0.93–1.06)	1.23 (1.16–1.30)	1.23 (1.17–1.28)

a Hazard ratio per year increase in age.

### Long-term survival as compared to the general population

Thirty-day survivors of ICH had a lower chance of survival than the general population over the entire 10-year period of follow-up (Table 4). As an example, relative survival after 5 years of 35 to 54 year olds was 0.90 (95% CI 0.88–0.92) in men and 0.91 (0.89–0.93) in women and that of 75 to 94 year olds 0.52 (0.49–0.55) in men and 0.52 (0.50–0.56) in women.

**Table 4.** Long-term survival of 30-day ICH survivors relative to the general Dutch population, matched for age, sex, and calendar year, at 1, 2, 5 and 10 years.

	1 year	2 years	5 years	10 years
Men 35–54 years	0.96 (0.95–0.97)	0.94 (0.93–0.95)	0.90 (0.88–0.92)	0.83 (0.80–0.87)
Women 35–54 years	0.97 (0.96–0.98)	0.95 (0.94–0.96)	0.91 (0.89–0.93)	0.86 (0.83–0.89)
Men 55–74 years	0.93 (0.92–0.94)	0.89 (0.88–0.90)	0.77 (0.75–0.78)	0.55 (0.52–0.58)
Women 55–74 years	0.94 (0.93–0.95)	0.89 (0.88–0.90)	0.77 (0.75–0.78)	0.60 (0.57–0.63)
Men 75–94 years	0.85 (0.84–0.87)	0.75 (0.73–0.77)	0.52 (0.49–0.55)	0.30 (0.25–0.36)
Women 75–94 years	0.84 (0.83–0.85)	0.75 (0.74–0.77)	0.52 (0.50–0.56)	0.28 (0.24–0.32)

## Discussion

Our study shows that ICH-survivors were not only at risk of recurrent ICH but also at similarly high risk of ischaemic stroke, both in men and women, in particular in the middle aged and in the elderly. Additionally, they had a sustained high risk of other vascular events and a reduced chance of survival compared to the general population up to 10 years after the ICH, in all age groups. Men had a higher risk of ischaemic stroke, of the composite vascular outcome and of death than women.

In multiple studies in Western Europe,^10,12–19^ North America,^20,21^ and Asia,^22–26^ one-year rates of recurrent ICH in ICH survivors were similar to the rates we found (0%–3.9%), whereas in three studies in Scotland,^27–29^ one in Italy,^30^ and one in Denmark,^31^ the rate of recurrent ICH was higher, around 6% to 9%/year (Supplementary Table e-5). The excess recurrent ICH in Scotland is unlikely due to methodological differences as two of the Scottish studies were record-linking studies and used ICD codes like we did in our study, and patients were of similar age.^27,28^ A recent study of health differences among regions in the UK showed that in Scotland the number of life-years lost and the number of disability-adjusted-life-years due to cerebrovascular diseases and ischaemic heart disease were higher than in other regions, probably due to variation in risk factors and socioeconomic deprivation.^32^ In the Italian cohort, collected between 1978 and 1982, poor blood pressure control, which was associated with recurrent ICH, may have contributed to the high ICH recurrence rate.^30^ The Danish cohort was record-linking like ours but included 7-day rather than 30-day survivors.^31^ Our finding that in the youngest age-group, the risk of ICH appeared higher than the risk of ischaemic stroke may be explained by a larger proportion of underlying macrovascular causes (e.g., arteriovenous malformation, aneurysm, cavernoma)^33^ and a lower prevalence of hypertension in this age-group compared to those of 55 years and older.^34^

For ischaemic stroke, the one-year rate varied among previous studies between 0% and 7.0% (Supplementary Table e-5),^10,13,16–21,24,27,28^ with again the highest rate in the Scottish population.^27^ The one-year risk of a composite vascular outcome was assessed in six previous studies in ICH survivors and varied between 1 and 25%/year,^10,13,21,27,28,35^ whereas in our study this risk was 4.3% in the youngest age-group and up to 14.6% in the elderly. In three studies, one from Scotland,^27^ one from Taiwan,^24^ and one from Japan,^36^ the reported 10-year risk of ICH varied between 9.6% and 13.7% (Supplementary Table e-5), which is higher than the 10-year rate we found (highest rate in women 55–74, 8.1%). We may have underestimated the rate of ICH, as in a substantial number of recurrent strokes it was not specified whether it was an ischaemic stroke or ICH. In the three studies, the 10-year risk of ischaemic stroke ranged from 6.9% to 12.9%,^24,27,36^ which is higher than the risk of ischaemic stroke we found but lower if we assume that a large proportion of the unspecified stroke in our study were ischaemic strokes. The reported 10-year risk of all stroke was 20.9% in Taiwan^24^ and 28.4% in Scotland,^27^ which was similar to the rates we found (22%–24% in the age-group of 55–74 years and 24%–26% in those 75–94 years). In contrast, one population-based study in Japan found a rate of 55.6% (95% CI 32.2–79.1%).^36^ However, the number of ICH patients in this cohort was small, resulting in imprecision.^36^ The cumulative risk of death after 10 years in our study of around 50% in the age-group 55 to 74 years was similar to that in a Swedish record-linking study,^17^ whereas the 10-year risk of death of around 20% in our youngest age-group was higher than the around 10% found in two previous studies in young adults,^37,38^ which may at least in part be explained by different age limits (35–54 in our youngest age-group, and 18–50^37^ or 16–49^38^ in the others; Supplementary Table e-5). The higher risk in men compared to women of ischaemic stroke of the composite vascular outcome and of death might be explained by a higher incidence of vascular disease in men in general or by differences in the treatment of hypertension and other vascular risk factors between men and women.

The finding that the total burden of future vascular events in ICH survivors is much larger than that of recurrent ICH alone has important implications for secondary preventive treatment for patients who survive ICH. Efforts to optimize outcome after ICH should not only be directed at the prevention of recurrent ICH but also at prevention of ischaemic events. In patients who experience ICH while on antiplatelets or anticoagulants, medication is often not restarted, which may add to the occurrence of ischaemic vascular complications after ICH. Evidence on the optimal secondary preventive treatment in these patients is limited and randomized controlled trials comparing restarting and avoiding antiplatelet- (RESTART and RESTART-Fr, NCT02966119; STATICH, NCT03186729) or anticoagulant drugs are needed and ongoing (APACHE-AF, NCT02565693;^39^ SoSTART, NCT03153150; NASPAF-ICH, NCT02998905; A3ICH, NCT03243175; and STATICH, NCT03186729; STROKECLOSE, NCT 02830152; PRESTIGE-AF, and ASPIRE). Recently, results of RESTART showed that in patients with ICH while on antithrombotic treatment, the risk of recurrent ICH with antiplatelet therapy, on average assigned 76 days after the ICH, is probably small and not exceeding the established benefits of antiplatelets for secondary prevention.^40^ In this study, there was no evidence of heterogeneity of the effect of antiplatelet therapy on the risk of recurrent ICH among pre-specified subgroups, including lobar versus non-lobar location of the ICH. A large observational study of the risk of death, stroke or functional outcome in patients with ICH while on anticoagulant treatment for atrial fibrillation suggested a benefit of restarting oral anticoagulants both in patients with non-lobar and in patients with lobar ICH.^41^ Another randomized controlled trial investigates the effect of more intense blood pressure control to prevent recurrent stroke in patients who have had an ICH (TRIDENT, NCT02699645).

Strengths of our study are that we were able to create a large population-based cohort of ICH survivors with long-term follow-up, that we not only studied the risk of recurrent ischaemic stroke and ICH but also of other vascular outcomes and that we assessed long-term survival relative to the general population. The size of the cohort enabled us to provide age- and sex specific estimates of risk at multiple time points. By correcting for competing risk of death, we were able to reduce overestimation of the risk of outcomes. Furthermore, the methods of linkage of the registries that we used have been validated,^7^ and we previously showed that the coding of ICH in the HDR is correct in 91% of cases.^6^ The reliability of ICD coding for ICH has also been demonstrated by others.^42^

Our study also has limitations. First, in a substantial proportion of patients, recurrent strokes, in particular those that were fatal, could not be classified as either ischaemic stroke or ICH. This hampers the accuracy of comparing the risk of recurrent ICH with that of ischaemic stroke. Nevertheless, based on the results for the combined vascular outcome and a high likelihood that among the unspecified strokes many are in fact ischaemic strokes,^43,44^ our data clearly point to a high risk of ischaemic events in addition to the risk of recurrent ICH. Second, during the inclusion period of the cohort, the participation in the HDR declined, leading to a possible underestimation of new non-fatal events in the years after 2004. Third, 15% of the Dutch population does not have a unique combination of sex, date of birth and four digit postal code and therefore could not be linked to the HDR and CDR. However, this percentage was stable during the study period, and it was previously demonstrated that there are no differences in cardiovascular disease between people with a unique identifier and those without.^45^ Therefore, we are confident that these missing persons have not influenced our results. Fourth, non-fatal events for which patients were not admitted to hospital were not included and may also have led to underestimation or recurrence rates. Fifth, it is possible that patients had a first ICH before the start of the cohort, which may have led to misclassification of a recurrent event as a first ICH. Sixth, in this registration-based study, we had no information on vascular risk factors other than represented by the CCI and no information about the use of secondary preventive medication. Finally, we had no information on the location of the ICH or its cause, which both affect recurrence rates of ICH and other outcomes.^3,19,29^

## Conclusion

Our large population-based record-linkage study shows that ICH survivors are not only at risk of recurrent ICH but also at risk of ischaemic stroke and other vascular events. The risk of ischaemic stroke appears to be at least as high as that of recurrent ICH. In addition, patients who survive ICH have a sustained reduced relative survival in all age groups compared to the general population. These findings warrant clinical trials to determine optimal secondary prevention treatment after ICH.

## Supplemental Material

sj-pdf-1-eso-10.1177_2396987320953394 - Supplemental material for Long-term prognosis after intracerebral haemorrhage Click here for additional data file. Supplemental material, sj-pdf-1-eso-10.1177_2396987320953394 for Long-term prognosis after intracerebral haemorrhage by Koen M van Nieuwenhuizen, Ilonca Vaartjes, Jamie I Verhoeven, Gabriel JE Rinkel, L Jaap Kappelle, Floris HBM Schreuder and Catharina JM Klijn in European Stroke Journal
