# Supplementary material for: Long-term prognosis after intracerebral haemorrhage
Source: Eur Stroke J. 2020 Sep 2;5(4):336–44. doi: 10.1177/2396987320953394 (PMC7856590; doi:10.1177/2396987320953394)
Supplement: sj-pdf-1-eso-10.1177_2396987320953394 - Supplemental material for Long-term prognosis after intracerebral haemorrhage [file sj-pdf-1-eso-10.1177_2396987320953394.pdf]

Table e-1: Participation rate of all Dutch hospitals in the Hospital Discharge Register (HDR)

Year	Percentage of hospitals participating in the HDR
1998	98.4%
1999	98.8%
2000	98.5%
2001	98.6%
2002	99.0%
2003	98.9%
2004	98.5%
2005	96.2%
2006	89.0%
2007	87.5%
2008	87.6%
2009	86.8%
2010	85.6%

Table e-2. ICD-9 and ICD-10 codes for the outcome events

	ICD-9		ICD-10
**Intracerebral haemorrhage**			
431	Intracerebral haemorrhage	I61	Intracerebral haemorrhage
**Ischaemic stroke**			
434	Occlusion of cerebral arteries	I63	Cerebral infarction
**Unspecified stroke**			
436	Acute, but ill-defined, cerebrovascular disease	I64	Stroke, not specified as haemorrhage or infarction
**All stroke**			
431	Intracerebral haemorrhage	I61	Intracerebral haemorrhage
434	Occlusion of cerebral arteries	I63	Cerebral infarction
436	Acute, but ill-defined, cerebrovascular disease	I64	Stroke, not specified as haemorrhage or infarction
430	Subarachnoid haemorrhage	I60	Subarachnoid haemorrhage
**Composite vascular outcome**			
410	Acute myocardial infarction	I21	Acute myocardial infarction
		I22	Subsequent myocardial infarction
		I23	Certain current complications following acute myocardial infarction
411	Other acute and subacute forms of ischaemic heart disease	I24	Other acute ischaemic heart diseases
413	Angina pectoris	I20	Angina pectoris
414	Chronic ischaemic heart disease	I25	Chronic ischaemic heart disease
415	Acute pulmonary heart disease	I27	Other pulmonary heart diseases
430	Subarachnoid haemorrhage	I60	Subarachnoid haemorrhage
431	Intracerebral haemorrhage	I61	Intracerebral haemorrhage
432	Other and unspecified intracranial haemorrhage	I62	Other and unspecified nontraumatic intracranial haemorrhage
434	Occlusion of cerebral arteries	I63	Cerebral infarction
436	Acute, but ill-defined, cerebrovascular disease	I64	Stroke, not specified as haemorrhage or infarction
441	Aortic dissection or aneurysm	I71	Aortic aneurysm and dissection
442	Arterial aneurysm (except aortic or intracranial)	I72	Other aneurysm and dissection
443	Other peripheral artery disease	I73	Other peripheral vascular disease
444	Arterial embolism and thrombosis	I26	Pulmonary embolism
444	Arterial embolism and thrombosis	I74	Arterial embolism and thrombosis
445	Atheroembolism	I75-I76	Arterial embolism and thrombosis
451	Phlebitis and thrombophlebitis	I80	Phlebitis and thrombophlebitis
452	Portal vein thrombosis	I81	Portal vein thrombosis
453	Venous embolism and thrombosis	I82	Other venous embolism and thrombosis

The Hospital Discharge Register contains reasons for admission coded with ICD-9. Causes of death are registered in the Cause of Death Register using ICD-10 codes.

Table e-3. Composition of the outcome all stroke

	**Deaths, n (%)**	**Admissions, n (%)**
All stroke*	1722	1655
Intracerebral haemorrhage	328 (19.0%)	449 (27.1%)
Cerebral infarction	72 (4.2%)	684 (41.3%)
Stroke, not specified as haemorrhage or infarction	1299 (75.4%)	466 (28.2%)
Subarachnoid haemorrhage	23 (1.3%)	56 (3.4%)

* numbers of the stroke subtypes as a component of the outcome all stroke may differ from the numbers reported for these outcomes separately as reported in the text because multiple events may occur in individual patients.

Table e-4. Composition of the composite vascular outcome

	**Deaths, n (%)**	**Admissions, n (%)**
**Composite vascular outcome**	2160	2805
Intracerebral haemorrhage	314 (14.5%)	418 (14.9%)
Cerebral infarction	69 (3.2%)	623 (22.2%)
Stroke, not specified as stroke or infarction	1269 (58.8%)	435 (15.5%)
Acute myocardial infarction	279 (12.9%)	295 (10.5%)
Other acute ischaemic heart disease	5 (0.2%)	175 (6.2%)
Aortic aneurysm and dissection	40 (1.9%)	137 (4.9%)
Angina pectoris	5 (0.2%)	168 (6.0%)
Pulmonary embolism	26 (1.1%)	117 (4.2%)
Chronic ischaemic heart disease	88 (4.1%)	43 (1.5%)
Arterial embolism and thrombosis	9 (0.4%)	116 (4.1%)
Other intracranial haemorrhage	15 (0.7%)	95 (3.4%)
Other venous embolism and thrombosis	1 (<0.1%)	87 (3.1%)
Subarachnoid haemorrhage	23 (1.1%)	52 (1.9%)
Other aneurysm and dissection	7 (0.3%)	29 (1.0%)
Phlebitis and thrombophlebitis	3 (0.1%)	15 (0.5%)
Other peripheral vascular diseases	4 (0.2%)	0
Acute pulmonary heart disease	3 (0.1%)	0

Table e-5. Summary of studies reporting on long term outcome (≥ 1 year) after intracerebral haemorrhage

Study	Country and period	Setting	Design	Population	Analytic method	Patients, n	Age, mean, y	Follow up duration, mean, y[a]	Intracerebral haemorrhage	Ischaemic stroke	All stroke	CVO	Vascular death	All death
**Douglas, 1982[1]**	United States 1975-1979	Two hospitals	Retrospective	Hypertensive ICH, clinical diagnosis, discharged alive	P	42	N/A	2.4	0 (0-5.8)[g]	N/A	N/A	4.8 (1.0-14.4)[g] [h]	7.1 (2.1-17.9)[g]	16.7 (7.8-30.0)[g]
**Helweg-Larsen, 1984[2]**	Denmark 1974-1982	Single hospital	Retrospective	Any ICH, clinical diagnosis, 21 days survivors	P	39	54 (median)	4.5 (median)	2.6 (0.3-11.4)[g]	N/A	5.1 (1.1-15.5)[g]	N/A	N/A	18.0 (8.4-32.0)[g]
**Fieschi, 1988[3]**	Italy 1982-1983	Single hospital	Retrospective	First ICH, clinical diagnosis, 30 days survivors	P	73	61.5[b]	1 (fixed)	0 (0-3.4)[g]	N/A	N/A	N/A	4.1 (1.2-10.6)[g]	5.5 (1.9-12.5)[g]
**Fogelholm, 1992[4]**	Finland 1985-1989	Population	Retrospective	First ICH, clinical diagnosis, 30 days survivors	P	79	68.2[b] (median)	2.7 (median)	7.6 (3.2-15.0)[g]	6.3 (2.5-13.3)[g]	13.9 (7.6-22.8)[g]	N/A	20.3 (12.6-30.1)[g]	30.4 (21.1-41.1)[g]
**Franke, 1992[5]**	Netherlands 1986-1989	Two hospitals	Prospective	First ICH, clinical diagnosis, 2 days survivors	P	120	N/A	1 (fixed)	N/A	N/A	3.3 (1.1-7.7)[g]	N/A	10.0 (5.6-16.3)[g]	38.8 (30.0-47.2)[g]
**Chen, 1995[6]**	Taiwan 1987-1989	Single hospital	Retrospective	Hypertensive ICH, clinical diagnosis, history of ICH	P	892	59	2.3 (median)	5.3 (4.0-6.9)[g]	N/A	N/A	N/A	N/A	N/A
**Counsell, 1995[7]**	United Kingdom 1981-1985	Population	Prospective	First ICH, clinical diagnosis, 30 days survivors	P<br>AR	31	68.9	2.5 (median)	12.9 (4.5-27.8)[g]	12.9 (4.5-27.8)[g]	29.0 (15.4-46.3)[g]<br>7.2 (3.4-13.8)[f]	N/A	19.4 (8.5-35.6)[g]	35.5 (20.5-53.0)[g]<br>8.9 (4.6-16.2)[f]
**Maruishi, 1995[8]**	Japan 1984-1992	Single hospital	Retrospective	Any ICH, clinical diagnosis, history of ICH	P	509	62.9	2.3	5.9 (4.1-8.2)[g]	N/A	N/A	N/A	N/A	N/A
**Passero, 1995[9]**	Italy 1978-1982	Single hospital	Prospective	First ICH, clinical diagnosis, 30 days survivors	P	112	63.7	7.0	24.1 (16.9-32.6)[g]<br>1 year risk:<br>7.1 (3.4-13.0)[g]<br>3 year risk:<br>14.3 (8.7-21.6)[g]<br>5 year risk:<br>18.6 (12.4-26.7)[g]	7.1 (3.4-13.0)[g]	31.3 (23.2-40.2)[g]	N/A	N/A	N/A
**Neau, 1997[10]**	France 1984-1994	Single hospital	Retrospective	First ICH, clinical diagnosis, 30 days survivors	P	375	64.7	4.0	6.4 (4.3-9.2)[g]	N/A	N/A	N/A	N/A	N/A
**Arakawa, 1998[11]**	Japan 1982-1996	Single hospital	Retrospective	First hypertensive ICH, clinical diagnosis, 3 months survivors	AR	74	59	5.6	2.0 (0.9-3.7)[f]	N/A	N/A	N/A	N/A	N/A
**Gonzalez-Duarte, 1998[12]**	Mexico 1989-1997	Single hospital	Retrospective	First ICH, clinical diagnosis, discharged alive	P	350	60	4.6	6.3 (4.1-9.2)[g]	N/A	N/A	N/A	N/A	N/A
**Hankey, 1998[13]**	Australia 1989-1990	Population	Prospective	First ICH, clinical diagnosis, 21 days survivors	P	36	73	5 (fixed)	8.3 (2.4-20.6)[g]	8.3 (2.4-20.6)[g]	27.8 (15.3-43.7)[g]	N/A	N/A	N/A
**Bae, 1999[14]**	Korea 1989-1995	Single hospital	Retrospective	Any hypertensive ICH, clinical diagnosis, 3 month survivors	P	617	58	3.2	8.4 (6.4-10.8)[g]<br>1 year risk:<br>2.8 (1.7-4.3)[g]<br>2 year risk:<br>5.5 (3.9-7.5)[g]<br>4 year risk:	N/A	N/A	N/A	N/A	N/A

									7.8 (5.9-10.1)[g]					
**Hill, 2000[15]**	Canada<br>1986-1996	Single hospital	Retrospective, record-linking	First ICH, clinical diagnosis, 30 days survivors	AR	172	65.7	3.6	2.4 (1.4-3.9)	3.0 (1.8-4.7)	N/A	N/A	N/A	N/A
**O'Donnel, 2000[16]**	United States<br>1994-1998	Single hospital	Prospective	Lobar ICH, > 55y clinical diagnosis, discharged alive	AR	71	75.4	2.0	13.4 (8.3-20.5)[f]	N/A	N/A	N/A	N/A	N/A
**Chen, 2001[17]**	Taiwan<br>1988-1999	Single hospital	Retrospective	Hypertensive ICH, clinical diagnosis, discharged alive	P	1,421	59.9	1.9 (median)	4.8 (3.8-6.0)[f]	N/A	N/A	N/A	N/A	N/A
**Vermeer, 2002[18]**	Netherlands<br>1986-1995	Three hospitals	Retrospective	First ICH, clinical diagnosis, returned home alive	AR<br>P	243	N/A	5.5	2.1 (1.4-3.3)	1.4 (0.8-2.3)	N/A	5.9 (4.5-7.7)[c]	3.2 (2.2-4.5)	-<br>5 year risk: 24% (18-30)[g]
**Hillen, 2003[19]**	United Kingdom<br>1995-2000	Population	Prospective	First ICH, clinical diagnosis, 21 days survivors	AR<br>P	220	71.4	1.2 (median)	2.3 (0.9-4.9)[f]	2.7 (1.1-5.5)[f]	1 year risk:<br>7.2 (3.8–13.4)<br>3 year risk:<br>11.1 (6.3–19.2)<br>5 year risk:<br>13.4 (7.6–22.9)	N/A	N/A	1 year risk:<br>50.1 (43.6–56.9)<br>3 year risk:<br>57.4 (50.7–64.3)<br>5 year risk:<br>67.4 (58.3–76.1)
**Yokota, 2004[20]**	Japan<br>1978-1997	Single hospital	Retrospective	First ICH, clinical diagnosis, direct after index ICH	P	350	61	3	3.4 (1.9-5.7)[g]	4.0 (2.3-6.4)[g]	7.4 (5.0-10.5)[g]	N/A	N/A	N/A
**Fogelholm, 2005[21]**	Finland<br>1985-1991	Population	Retrospective	First ICH, clinical diagnosis, 28 days survivors	P	203	66.8	6.1 (median)	11.3 (7.5-16.2)[g]	10.3 (6.7-15.1)[g]	35.0 (28.7-41.7)[g]	N/A	45.3 (38.6-52.2)[g]	78.3 (72.3-83.6)[g]
**Hata, 2005[22]**	Japan<br>1961-1993	Population	Prospective	First ICH, >40y clinical diagnosis, direct after index ICH	P	73	N/A	10 (fixed)	9.6 (4.4-17.9)[g]	6.9 (2.7-14.4)[g]	1 year risk:<br>25.6 (9.0-42.2)<br>5 year risk:<br>34.9 (16.0-53.8)<br>10 year risk:<br>55.6 (32.2-79.1)	N/A	N/A	N/A
**Inagawa, 2005[23]**	Japan<br>1991-1998	Population	Prospective	First ICH, clinical diagnosis, discharged alive	AR	279	63.3	3	2.3 (1.4-3.5)[f]	N/A	N/A	N/A	N/A	N/A
**Saloheimo, 2006[24]**	Finland<br>1993-1995	Single hospital	Prospective	First ICH, clinical diagnosis, 3 months survivors	AR<br>P	140	65.9	7 (fixed)	1.3 (0.7-2.3)[f,h]	N/A	N/A	N/A	2.7 (1.7-4.0)[f]<br>7 year risk:<br>15.7 (10.4-22.4)[g]	5.6 (4.2-7.4)[f]<br>7 year risk:<br>32.9 (25.5-40.9)[g]
**Hanger, 2007[25]**	New Zealand<br>1996-2004	Three hospitals	Retrospective	First ICH, ICD code based, discharged alive	P	453	72.4[b]	N/A	4.2 (2.6-6.3)[g]	3.8 (2.3-5.8)[g]	7.5 (5.4-10.2)[g]	N/A	N/A	32.3 (28.1-36.6)[g]
**McGuire, 2007[26]**	Scotland<br>1995	All hospitals in Scotland	Retrospective, record-linking	ICH, no stroke in prior 5y, ICD code based, 30 days survivors	P	705	65[b]	11 (fixed)	1 year risk:<br>8.0 (5.4-10.6)<br>5 year risk:<br>12.7 (9.5-15.9)<br>10 year risk:<br>13.7 (10.4-16.9)	1 year risk:<br>7.0 (4.5-9.5)<br>5 year risk:<br>11.1 (7.8-14.5)<br>10 year risk:<br>12.9 (9.3-16.6)	1 year risk:<br>14.8 (12.7-19.7)<br>5 year risk:<br>23.6 (20.4-28.8)<br>10 year risk:<br>28.4 (24.0-32.8)	1 year risk:<br>1.5 (0.4-2.7)[i]<br>5 year risk:<br>3.6 (1.8-5.4)[i]<br>10 year risk:<br>7.0 (4.5-9.5)[i]	N/A	N/A
**Lee, 2007[27]**	United States<br>1997	5% of Medicare database	Retrospective, record-linking	First ICH, >64y ICD code based, discharged alive	P	1,457	N/A	4 (fixed)	4 year risk:<br>5.1 (4.2-6.2)[g]	4 year risk:<br>7.1 (6.0-8.3)[g]	4 year risk:<br>8.9 (7.7-10.2)[g]	N/A	N/A	N/A
**Yen, 2007[28]**	Taiwan<br>1999-2003	Single hospital	Retrospective	Any ICH, clinical diagnosis unknown intercept	P	585	64	2.8	5.8 (4.1-7.9)[g]	N/A	N/A	N/A	N/A	16.1 (13.3-19.2)[g]

**Christensen, 2008[29]**	Scotland<br>2004-2005	All hospitals in Scotland	Retrospective, record-linking	ICH, no stroke in prior 5y, ICD code based, discharged alive	P	557	67.6[b]	1 (fixed)	1 year risk:<br>8.4 (6.0-10.8)	1 year risk:<br>1.9 (0.7-3.1)	1 year risk:<br>15.0 (12.1-17.9)	1 year risk:<br>1.0 (0.2-1.8)[i]	N/A	1 year risk:<br>7.3 (5.6-8.9)
**Azarpazhooh, 2008[30]**	Australia<br>1996-1999	Population	Prospective	First ICH, >64y clinical diagnosis, 28 days survivors	P	191	N/A	2 (fixed)	2.6 (1.0-5.6)[g]	3.7 (1.7-7.1)[g]	6.3 (3.5-10.4)[g]	N/A	N/A	N/A
**Zia, 2009[31]**	Sweden<br>1993-2000	Single hospital	Prospective	First ICH, clinical diagnosis, 28 days survivors	AR<br>P	353	73[b]	3 (fixed)	2.3 (1.4-3.5)[f]<br>3 year risk:<br>5.7 (3.6-8.5)[g]	2.8 (1.8-4.0)[f]<br>3 year risk:<br>6.8 (4.5-9.8)[g]	5.1 (3.7-6.7)[f]<br>3 year risk:<br>12.5 (9.3-16.2)[g]	N/A	-<br>3 year risk:<br>18.7 (14.9-23.0)[g]	-<br>3 year risk:<br>30.3 (25.7-35.3)[g]
**Weimar, 2011[32]**	Germany<br>2002-2006	13 hospitals	Prospective	First ICH, clinical diagnosis, discharged alive	P<br><br>AR	496	69.6 [b]	2	2.2 (1.2-3.8)[g]	4.2 (2.7-6.3)[g]	9.3 (7.0-12.1)[g]<br><br>4.5 (3.1-5.9)	N/A	6.3 (4.4-8.6)[g]	21.6 (18.1-25.4)[g]<br>8.5 (6.9-10.1)
**Hansen, 2013[33]**	Sweden<br>1996	12 hospitals	Retrospective, record-linking	First ICH, clinical diagnosis, 1 year survivors	AR<br>P	172	67.7	13 (fixed)	0.5 (0.2-1.1)[f h]	0.7 (0.3-1.3)[f h]	3.2 (2.4-4.3)[f h]	N/A	4.9 (3.8-6.2)[f]	8.8 (7.3-10.6)[f]<br>5 year risk:<br>26.2 (20.0-33.1)[g]<br>10 year risk:<br>57.0 (49.5-64.2)[g]
**Jones, 2013[34]**	United States	Population	Prospective	First ICH, clinical diagnosis, no intercept	AR	85	66	5.3 (median)	1.1 (0.3-2.9)[f]	2.1 (0.9-4.4)[f]	3.2 (1.6-5.8)[f]	9.4 (3.6-23.2)	N/A	42.8 (30.6-57.4)
**Rutten-Jacobs, 2013[35]**	Netherlands<br>1980-2010	Single hospital	Prospective	First ICH, age 18-50y clinical diagnosis, 30 days survivors	AR<br>P	71	38.1	6.1 (median)	0.3 (0-0.9)[f h]	0 (0-0.4)[f h]	0.3 (0-0.9)[f h]	N/A	0.8 (0.3-1.6)[f]	1.1 (0.6-2.2)[f]<br>1 year risk:<br>2.9 (0.0-6.8)<br>5 year risk:<br>6.1 (0.3-11.9)<br>10 year risk:<br>10.3 (2.3-18.3)<br>20 year risk:<br>13.7 (3.6-23.9)
**Rutten-Jacobs, 2013[36]**	Netherlands<br>1980-2010	Single hospital	Prospective	First ICH, age 18-50y clinical diagnosis, 30 days survivors	P	68	38.0	6.6	5.9 (2.0-13.4)[g]	1.5 (0.2-6.7)[g]	7.4 (2.9-15.4)[g]	8.8 (3.8-17.3)[g d]	N/A	N/A
**Pennlert, 2014[37]**	Sweden<br>1995-2008	Population	Prospective	First ICH, age 25-74y clinical diagnosis, 28 days survivors	P	815	62.1	4.0	4.1 (2.9-5.6)[g]	7.7 (6.1-9.7)[g]	12.4 (10.3-14.8)[g]	N/A	N/A	N/A
**Yeh, 2014[38]**	Taiwan<br>1995-2013	Single hospital	Prospective	First ICH, clinical diagnosis, 1 month survivors	P	3,785	58.7 [b]	5.5	1 year risk:<br>3.2 (2.7-3.8)<br>5 year risk:<br>7.7 (6.9-8.6)<br>10 year risk:<br>12.9 (11.9-14.0)	1 year risk:<br>1.4 (1.1-1.8)<br>5 year risk:<br>4.7 (4.1-5.4)<br>10 year risk:<br>10.2 (9.3-11.2)	1 year risk:<br>3.6 (3.0-4.2)<br>5 year risk:<br>11.6 (10.6-12.7)<br>10 year risk: 20.9 (19.6-22.2)	N/A	N/A	1 year risk:<br>26.3 (24.9-27.7)[g]
**Biffi, 2015[39]**	United States<br>1994-2013	Single hospital	Prospective	Any ICH, clinical diagnosis, 90 days survivors	P	1,145	73.4	3.1 (median)	12.8 (10.9-14.8)[g]	N/A	N/A	N/A	N/A	N/A
**Koivunen, 2015[40]**	Finland,<br>2000-2010	Single hospital	Retrospective, record-linking	First ICH, age 16-49y clinical diagnosis, 30 days survivors	P	131[e]	N/A	9.7 (median)	7.6 (4.0-13.1)[g]	3.1 (1.0-7.1)[g]	10.7 (6.3-16.8)[g]	N/A	N/A	1 year risk:<br>1.9 (0.0-3.8)<br>5 year risk:<br>6.8 (2.9-10.7)<br>10 year risk:<br>11.2 (7.3-15.1)
**Samarasekera, 2015[41]**	Scotland<br>2010-2011	Population	Prospective	First ICH clinical diagnosis, no intercept	P<br>AR	128	78 (median)	1 (fixed)	3.1 (1.1-7.3)[g]<br>6.3 (2.0-15.3)[f]	N/A	N/A	N/A	N/A	56.3 (47.6-64.6)[g]
**Bjerkreim, 2016[42]**	Norway<br>2007-2012	Single hospital	Prospective	First ICH, clinical diagnosis, discharged alive	P	121	72.3	1 (fixed)	1.4 (0.2-6.3)[g]	2.8 (0.6-8.6)[g]	4.2 (1.2-10.7)[g]	9.7 (4.5-18.1)[g]	N/A	14.9 (8.9-22.7)[g]

**Callaly, 2016**[43]	Ireland 2006	Population	Prospective	Any ICH, clinical diagnosis, 28 days survivors	P	61	70.6	2 (fixed)	3.3 (0.7-10.1)[g]	0 (0.0-4.0)[g]	3.3 (0.7-10.1)[g]	N/A	N/A	6.7 (1.7-24.1)
**Ottosen, 2016**[44]	Denmark 2005-2013	Nationwide	Retrospective, record-linking	First ICH, age >18y, ICD code based, 30 days survivors	P	6,369	N/A	2.3 (median)	3.2 (2.8-3.7)[g]	N/A	N/A	N/A	N/A	N/A
**Schmidt, 2016**[45]	Denmark 1996-2011	Nationwide	Retrospective, record-linking	First ICH, age >20y, ICD code based, 7 days after discharge	P	15,270	N/A	2.8 (median)	1 year risk: 8.9 (8.4-9.3) 5 year risk: 13.7 (13.2-14.3)	N/A	N/A	N/A	N/A	N/A
**Wolf, 2016**[46]	Germany 1998-2014	Single hospital	Retrospective	First ICH, clinical diagnosis, 28 days survivors	P	1,273	69	1.5 (median)	2.6 (1.8-3.6)[g]	N/A	N/A	N/A	N/A	N/A
**He, 2017**[47]	China 2007-2008	109 hospitals	Retrospective	Any ICH, ICD code based, unknown intercept	P	23,748	58	2 (fixed)	3.3 (3.0-6.5)[g]	2.1 (1.9-2.2)[g]	5.4 (5.1-5.7)[g]	N/A	N/A	N/A
**Qiu, 2017**[48]	Singapore 2006-2013	Single hospital	Prospective	First ICH, clinical diagnosis, 14 days survivors	AR P	1,708	62	3.8	1.1 (0.8-1.3)[f] 3.5 (2.7-4.5)[g]	N/A	N/A	N/A	N/A	8.7 (8.0-9.5)[f] 32.6 (30.4-34.9)[g]
**Tsivgoulis, 2018**[49]	Greece 2010-2012	Population	Prospective	First ICH, clinical diagnosis, 1 day survivors	P	83	77	1 (fixed)	N/A	N/A	1.2 (0-7.2)	N/A	N/A	47.0 (36.6-57.6)
**Casolla 2019**[50]	France 2004-2009	Single hospital	Prospective	Any ICH, clinical diagnosis, 30-day survivors	P	310	70 (median)	6 (median)	1-year risk: 3.9 (2.1-6.5) 5-year risk: 4.9 (2.9-7.7)	1-year risk: 3.2 (1.7-5.7) 5-year risk: 9.0 (6.1-12.6)	1-year risk: 7.1 (4.6-18.4) 5-year risk: 14.2 (10.5-18.4)	N/A	N/A	N/A

Abbreviations: AR, annual rate; N/A, not available; P, proportion.

[a] Unless otherwise specified

[b] Mean age relates to the whole cohort, including non-survivors

[c] Defined as stroke, myocardial infarction, or major extracranial haemorrhage

[d] Defined as the composite event of fatal or non-fatal stroke (ischaemic or hemorrhagic), fatal or nonfatal myocardial infarction, or cardiovascular procedures (coronary artery bypass grafting, percutaneous transluminal coronary angioplasty, carotid endarterectomy, or other peripheral arterial revascularization procedures), whichever occurred first

[e] All-cause mortality was available for 282 patients

[f] 95%-CI calculated by Mid-P exact test

[g] 95%-CI calculated by Jeffreys score interval

[h] Only fatal events

[i] Defined as fatal or non-fatal acute myocardial infarction, fatal arrhythmia, fatal heart failure and fatal cardiac arrest.

[j] Defined as the composite of stroke, acute coronary syndrome, acute limb ischemia, coronary artery revascularization (either coronary artery bypass or percutaneous coronary intervention), severe peripheral artery disease (ie, symptomatic arterial stenosis or stenosis requiring a revascularization procedure), and severe internal carotid stenosis requiring a revascularization procedure, bleeding with hemoglobin drop of ≥3g/dL or needing blood transfusion, surgical intervention or intravenous vasoactive agents, imaging confirmed intracranial or intraspinal haemorrhage or intraocular bleed, coronary bypass-related bleeding, or fatal bleeding

Supplementary references

1. Douglas MA, Haerer AF. Long-term prognosis of hypertensive intracerebral hemorrhage. Stroke; a journal of cerebral circulation 1982;13:488-491.
2. Helweg-Larsen S, Sommer W, Strange P, Lester J, Boysen G. Prognosis for patients treated conservatively for spontaneous intracerebral hematomas. Stroke 1984;15:1045-1048.
3. Fieschi C, Carolei A, Fiorelli M, et al. Changing prognosis of primary intracerebral hemorrhage: results of a clinical and computed tomographic follow-up study of 104 patients. Stroke; a journal of cerebral circulation 1988;19:192-195.
4. Fogelholm R, Nuutila M, Vuorela AL. Primary intracerebral haemorrhage in the Jyvaskyla region, central Finland, 1985-89: incidence, case fatality rate, and functional outcome. J Neurol Neurosurg Psychiatry 1992;55:546-552.
5. Franke CL, van Swieten JC, Algra A, van Gijn J. Prognostic factors in patients with intracerebral haematoma. J Neurol Neurosurg Psychiatry 1992;55:653-657.
6. Chen ST, Chiang CY, Hsu CY, Lee TH, Tang LM. Recurrent hypertensive intracerebral hemorrhage. Acta neurologica Scandinavica 1995;91:128-132.
7. Counsell C, Boonyakarnkul S, Dennis M, et al. Primary intracerebral hemorrhage in the Oxfordshire Community Stroke Project. Cerebrovascular diseases 1995;5:26-34.
8. Maruishi M, Shima T, Okada Y, Nishida M, Yamane K, Okita S. Clinical findings in patients with recurrent intracerebral hemorrhage. Surgical neurology 1995;44:444-449.
9. Passero S, Burgalassi L, D'Andrea P, Battistini N. Recurrence of Bleeding in Patients With Primary Intracerebral Hemorrhage. Stroke; a journal of cerebral circulation 1995;26:1189-1192.
10. Neau JP, Ingrand P, Couderq C, et al. Recurrent intracerebral hemorrhage. Neurology 1997;49:106-113.
11. Arakawa S, Saku Y, Ibayashi S, Nagao T, Fujishima M. Blood pressure control and recurrence of hypertensive brain hemorrhage. Stroke 1998;29:1806-1809.
12. Gonzalez-Duarte A, Cant C, Ruz-Sandoval JL, Barinagarrementeria F. Recurrent primary cerebral hemorrhage: frequency, mechanisms, and prognosis. Stroke; a journal of cerebral circulation 1998;29:1802-1805.
13. Hankey GJ, Jamrozik K, Broadhurst RJ, et al. Long-term risk of first recurrent stroke in the Perth Community Stroke Study. Stroke; a journal of cerebral circulation 1998;29:2491-2500.
14. Bae HG, Jeong DS, Doh JW, Lee KS, Yun IG, Byun BJ. Recurrence of bleeding in patients with hypertensive intracerebral hemorrhage. Cerebrovascular diseases 1999;9:102-108.
15. Hill MD, Silver FL, Austin PC, Tu JV. Rate of stroke recurrence in patients with primary intracerebral hemorrhage. Stroke 2000;31:123-127.
16. O'Donnell HC, Rosand J, Knudsen KA, et al. Apolipoprotein E genotype and the risk of recurrent lobar intracerebral hemorrhage. The New England journal of medicine 2000;342:240-245.
17. Chen CH, Huang CW, Chen HH, Lai ML. Recurrent hypertensive intracerebral hemorrhage among Taiwanese. Kaohsiung J Med Sci 2000;17:556-563.
18. Vermeer SE, Algra A, Franke CL, Koudstaal PJ, Rinkel GJ. Long-term prognosis after recovery from primary intracerebral hemorrhage. Neurology 2002;59:205-209.
19. Hillen T, Coshall C, Tilling K, et al. Cause of stroke recurrence is multifactorial: patterns, risk factors, and outcomes of stroke recurrence in the South London Stroke Register. Stroke; a journal of cerebral circulation 2003;34:1457-1463.
20. Yokota C, Minematsu K, Hasegawa Y, Yamaguchi T. Long-term prognosis, by stroke subtypes, after a first-ever stroke: a hospital-based study over a 20-year period. Cerebrovascular diseases 2004;18:111-116.
21. Fogelholm R, Murros K, Rissanen A, Avikainen S. Long term survival after primary intracerebral haemorrhage: a retrospective population based study. J Neurol Neurosurg Psychiatry 2005;76:1534-1538.
22. Hata J, Tanizaki Y, Kiyohara Y, et al. Ten year recurrence after first ever stroke in a Japanese community: the Hisayama study. J Neurol Neurosurg Psychiatry 2005;76:368-372.

23. Inagawa T. Recurrent primary intracerebral hemorrhage in Izumo City, Japan. Surg Neurol 2005;64:28-35; discussion 35-26.
24. Saloheimo P, Lapp TM, Juvela S, Hillbom M. The impact of functional status at three months on long-term survival after spontaneous intracerebral hemorrhage. Stroke 2006;37:487-491.
25. Hanger HC, Wilkinson TJ, Fayez-Iskander N, Sainsbury R. The risk of recurrent stroke after intracerebral haemorrhage. J Neurol Neurosurg Psychiatry 2007;78:836-840.
26. McGuire AJ, Raikou M, Whittle I, Christensen MC. Long-term mortality, morbidity and hospital care following intracerebral hemorrhage: an 11-year cohort study. Cerebrovasc Dis 2007;23:221-228.
27. Lee WC, Joshi AV, Wang Q, Pashos CL, Christensen MC. Morbidity and mortality among elderly Americans with different stroke subtypes. Adv Ther 2007;24:258-268.
28. Yen CC, Lo YK, Li JY, Lin YT, Lin CH, Gau YY. Recurrent primary intracerebral hemorrhage: a hospital based study. Acta Neurol Taiwan 2007;16:74-80.
29. Christensen MC, Munro V. Ischemic stroke and intracerebral hemorrhage: the latest evidence on mortality, readmissions and hospital costs from Scotland. Neuroepidemiology 2008;30:239-246.
30. Azarpazhooh MR, Nicol MB, Donnan GA, et al. Patterns of stroke recurrence according to subtype of first stroke event: the North East Melbourne Stroke Incidence Study (NEMESIS). International journal of stroke : official journal of the International Stroke Society 2008;3:158-164.
31. Zia E, Engstrom G, Svensson PJ, Norrving B, Pessah-Rasmussen H. Three-year survival and stroke recurrence rates in patients with primary intracerebral hemorrhage. Stroke 2009;40:3567-3573.
32. Weimar C, Benemann J, Terborg C, et al. Recurrent stroke after lobar and deep intracerebral hemorrhage: a hospital-based cohort study. Cerebrovascular diseases 2011;32:283-288.
33. Hansen BM, Nilsson OG, Anderson H, Norrving B, Saveland H, Lindgren A. Long term (13 years) prognosis after primary intracerebral haemorrhage: a prospective population based study of long term mortality, prognostic factors and causes of death. J Neurol Neurosurg Psychiatry 2013;84:1150-1155.
34. Jones SB, Sen S, Lakshminarayan K, Rosamond WD. Poststroke outcomes vary by pathogenic stroke subtype in the Atherosclerosis Risk in Communities Study. Stroke 2013;44:2307-2310.
35. Rutten-Jacobs LC, Arntz RM, Maaijwee NA, et al. Long-term mortality after stroke among adults aged 18 to 50 years. Jama 2013;309:1136-1144.
36. Rutten-Jacobs LC, Maaijwee NA, Arntz RM, et al. Long-term risk of recurrent vascular events after young stroke: The FUTURE study. Ann Neurol 2013;74:592-601.
37. Pennlert J, Eriksson M, Carlberg B, Wiklund PG. Long-term risk and predictors of recurrent stroke beyond the acute phase. Stroke; a journal of cerebral circulation 2014;45:1839-1841.
38. Yeh SJ, Tang SC, Tsai LK, Jeng JS. Pathogenetical subtypes of recurrent intracerebral hemorrhage: designations by SMASH-U classification system. Stroke 2014;45:2636-2642.
39. Biffi A, Anderson CD, Battey TW, et al. Association Between Blood Pressure Control and Risk of Recurrent Intracerebral Hemorrhage. Jama 2015;314:904-912.
40. Koivunen RJ, Tatlisumak T, Satopaa J, Niemela M, Putaala J. Intracerebral hemorrhage at young age: long-term prognosis. Eur J Neurol 2015;22:1029-1037.
41. Samarasekera N, Fonville A, Lerpiniere C, et al. Influence of intracerebral hemorrhage location on incidence, characteristics, and outcome: population-based study. Stroke 2015;46:361-368.
42. Bjerkreim AT, Thomassen L, Waje-Andreassen U, Selvik HA, Naess H. Hospital Readmission after Intracerebral Hemorrhage. J Stroke Cerebrovasc Dis 2016;25:157-162.
43. Callaly E, Ni Chroinin D, Hannon N, et al. Rates, Predictors, and Outcomes of Early and Late Recurrence After Stroke: The North Dublin Population Stroke Study. Stroke; a journal of cerebral circulation 2016;47:244-246.
44. Ottosen TP, Grijota M, Hansen ML, et al. Use of Antithrombotic Therapy and Long-Term Clinical Outcome Among Patients Surviving Intracerebral Hemorrhage. Stroke; a journal of cerebral circulation 2016;47:1837-1843.

45. Schmidt LB, Goertz S, Wohlfahrt J, Melbye M, Munch TN. Recurrent Intracerebral Hemorrhage: Associations with Comorbidities and Medicine with Antithrombotic Effects. PLoS One 2016;11:e0166223.
46. Wolf ME, Alonso A, Ebert AD, Szabo K, Chatzikonstantinou A. Etiologic and Clinical Characterization of Patients with Recurrent Spontaneous Intracerebral Hemorrhage. European neurology 2016;76:295-301.
47. He Q, Wu C, Guo W, et al. Hospital-Based Study of the Frequency and Risk Factors of Stroke Recurrence in Two Years in China. Journal of stroke and cerebrovascular diseases : the official journal of National Stroke Association 2017;26:2494-2500.
48. Qiu L, Upadhyaya T, See AA, Ng YP, Kon Kam King N. Incidence of Recurrent Intracerebral Hemorrhages in a Multiethnic South Asian Population. J Stroke Cerebrovasc Dis 2017;26:666-672.
49. Tsivgoulis G, Katsanos AH, Patousi A, et al. Stroke recurrence and mortality in northeastern Greece: the Evros Stroke Registry. Journal of neurology 2018;265:2379-2387.
50. Casolla B, Moulin S, Kyheng M, et al. Five-Year Risk of Major Ischemic and Hemorrhagic Events After Intracerebral Hemorrhage. Stroke 2019;50:1100-1107.
